# Comparing the return on investment of technologies to detect substandard and falsified amoxicillin: A Kenya case study

**DOI:** 10.1371/journal.pone.0268661

**Published:** 2023-01-18

**Authors:** Colleen R. Higgins, Betty Kobia, Sachiko Ozawa

**Affiliations:** 1 Division of Practice Advancement and Clinical Education, University of North Carolina Eshelman School of Pharmacy, University of North Carolina, Chapel Hill, North Carolina, United States of America; 2 Department of Maternal and Child Health, University of North Carolina Gillings School of Global Public Health, University of North Carolina, Chapel Hill, North Carolina, United States of America; Universidad Nacional Autonoma de Nicaragua Leon, NICARAGUA

## Abstract

The prevalence of substandard and falsified medicines in low- and middle-income countries (LMICs) is a major global public health concern. Multiple screening technologies for post-market surveillance of medicine quality have been developed but there exists no clear guidance on which technology is optimal for LMICs. This study examined the return on investment (ROI) of implementing a select number of screening technologies for post-market surveillance of amoxicillin quality in a case study of Kenya. An agent-based model, Examining Screening Technologies using Economic Evaluations for Medicines (ESTEEM), was developed to estimate the costs, benefits, and ROI of implementing screening technologies for post-market surveillance of substandard and falsified amoxicillin for treatment of pediatric pneumonia in Kenya. The model simulated sampling, testing, and removal of substandard and falsified amoxicillin from the Kenyan market using five screening technologies: (1) Global Pharma Health Fund’s GPHF-Minilab, (2) high-performance liquid chromatography (HPLC), (3) near-infrared spectroscopy (NIR), (4) paper analytical devices / antibiotic paper analytical devices (PADs/aPADs), and (5) Raman spectroscopy. The study team analyzed the population impact of utilizing amoxicillin for the treatment of pneumonia in children under age five in Kenya. We found that the GPHF-Minilab, NIR, and PADs/aPADs were similar in their abilities to rapidly screen for and remove substandard and falsified amoxicillin from the Kenyan market resulting in a higher ROI compared to HPLC. NIR and PADs/aPADs yielded the highest ROI at $21 (90% Uncertainty Range (UR) $5-$51) each, followed by GPHF-Minilab ($16, 90%UR $4 - $38), Raman ($9, 90%UR $2 - $21), and HPLC ($3, 90%UR $0 - $7). This study highlights screening technologies that can be used to reduce costs, speed up the removal of poor-quality medicines, and consequently improve health and economic outcomes in LMICs. National medicine regulatory authorities should adopt these fast, reliable, and low-cost screening technologies to better detect substandard and falsified medicines, reserving HPLC for confirmatory tests.

## Introduction

The use of substandard and falsified medical products has far-reaching health and economic implications. The World Health Organization (WHO) defines substandard medical products as authorized products that fail to meet either their quality standards, their specifications, or both [1]. Falsified medical products are those that deliberately or fraudulently misrepresent their identity, composition, or source [1]. At the individual level, consumption of these medicines can lead to clinical consequences such as treatment failure that prolongs illness [2]. In worst cases, this can potentially lead to harmful side effects or even death [2]. These medicines can promote antimicrobial resistance, undermine confidence in healthcare professionals, and increase medical costs for families and health systems [1–5]. WHO estimates 10.5% of medicines in low- and middle-income countries (LMICs) are substandard or falsified [1]. The estimated cost of substandard and falsified drugs across LMICs ranges from $10 billion to $200 billion, representing a large burden on resource-limited countries [3].

Antibiotics and antimalarials are among the most common classes of drugs reported as substandard and falsified, posing a risk for antimicrobial resistance and poor health outcomes [1,3,6]. A 2018 meta-analysis study showed that 12.4% of antibiotics tested in Africa and Asia were falsified or substandard [3]. Amoxicillin is the standard antibiotic of choice for the treatment of childhood pneumonia in Kenya [7,8]. The burden of pneumonia in Kenya is relatively high with an estimated annual incidence of 402 cases per 1000 children under age five between 2000 and 2015 [9]. Thus, ensuring the quality of amoxicillin treatments available to Kenyan children is important.

The proliferation of substandard and falsified medicines has fueled the development of a variety of technologies used to screen for poor-quality products [10–12]. These technologies vary in specificity, sensitivity, cost, portability, labor, and infrastructural requirements which determine their feasibility in LMICs [10]. High-performance liquid chromatography (HPLC), a sophisticated analytical technology, is regarded as the gold standard for determining a medication’s active pharmaceutical ingredients (API) in compendial analysis [13]. However, HPLC is costly, time-consuming, and requires highly specialized technicians and laboratory infrastructure to operate, rendering it less reliable for routine detection of poor-quality medicines in LMICs [10]. Using less expensive screening technologies to detect substandard and falsified medicines first, then using HPLC for confirmation is a strategy to save money and screen medicines more quickly [14]. For example, state regulatory authorities can deploy mobile testing vans equipped with portable screening devices to screen medicines across geographical areas and utilize those results to trigger enforcement actions to remove poor-quality medicines [15].

A previous review examined 42 qualitative and quantitative screening technologies and assigned them suitability scores for use in LMICs based on level of personnel training, electricity requirement, and speed, among other factors [10]. The Paper Analytical Devices (PADs) and antibiotic Paper Analytical Devices (aPADs), Thin Layer Chromatography Global Pharma Health Fund’s (GPHF) GPHF-Minilab, Near-Infrared (NIR), and Raman spectroscopies were some of the notable low-to-medium cost (USD <$100,000) screening technologies identified in the study as medium to highly suitable for use in LMICs [10]. In another study, 12 portable devices were evaluated for field-testing, and six of them were deemed suitable for use in Laos based on their ease of use, ease of training, ease of export, portability, and minimal consumables [16]. The six devices included PADs, GPHF-Minilab, two Raman spectrometers, and two NIR spectrometers [16]. However, as more screening devices become available, there exists little guidance when choosing between these technologies.

The expected return on investing in a particular screening technology is an important consideration for regulatory authorities. In a previous case study, we analyzed the cost-savings of utilizing PADs/aPADs to screen for substandard and falsified amoxicillin in Kenya [14]. However, to our knowledge, no study has analyzed the return on investment comparing screening technologies in LMICs. This study estimates the return on investment of implementing HPLC, PADs/aPADs, GPHF-Minilab, NIR, and TruScan Raman spectroscopy in surveillance of antibiotic quality in Kenya. The results of this analysis are aimed at informing decision-making for national regulatory bodies and may serve as an example for other LMICs on optimal post-market surveillance strategies.

## Methods

This is a return-on-investment study. The study team developed an agent-based model, Examining Screening Technologies using Economic Evaluations for Medicines (ESTEEM), to estimate the costs, benefits, and return on investment of implementing screening technologies for post-market surveillance of substandard and falsified amoxicillin in Kenya. The ESTEEM model has been piloted and validated in a previous study, where detailed methods can be found [14]. A literature search was conducted on existing technologies used to detect poor-quality medicines in LMICs. Five technologies that are relevant in LMICs were identified: PADs/aPADs, GPHF-Minilab, NIR, TruScan Raman spectroscopy, and HPLC [10–12,17].

**Fig 1:**
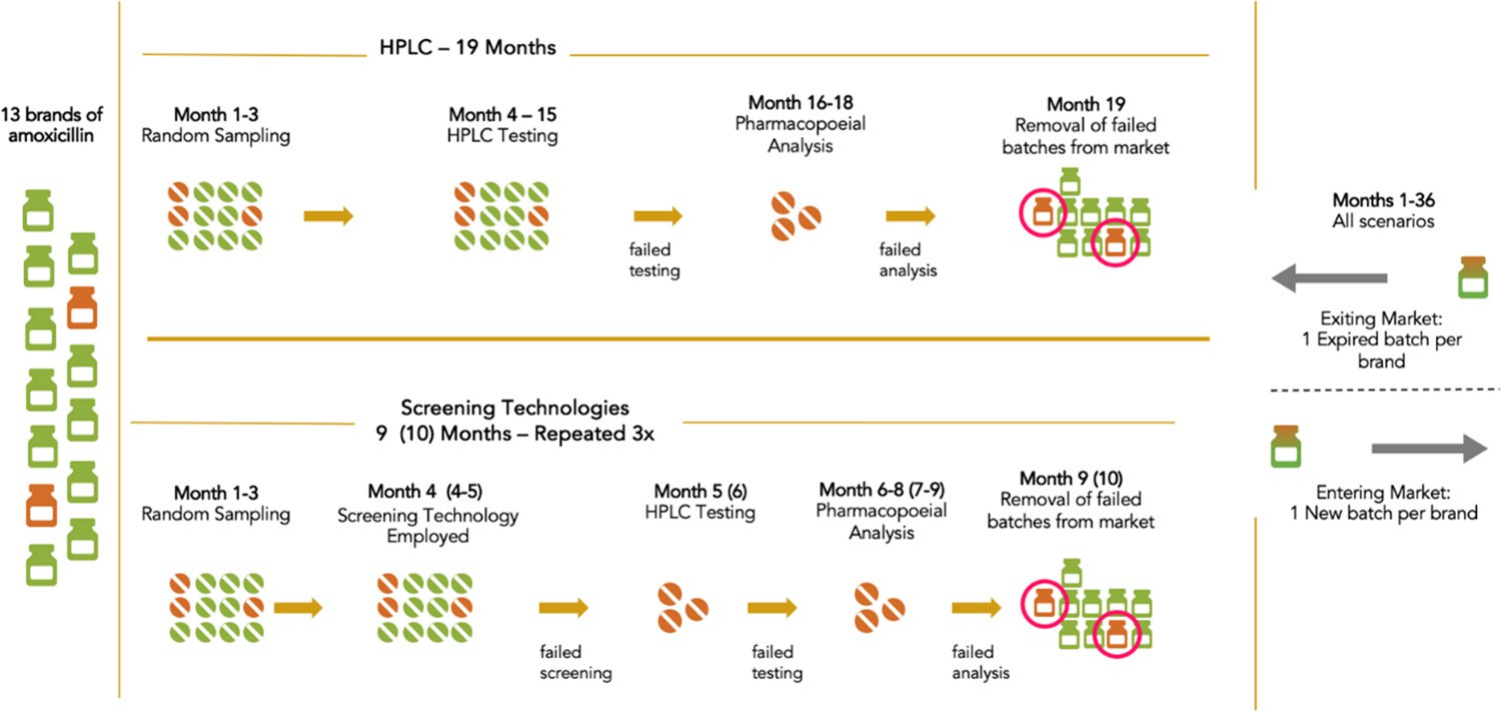
Flow diagram of the ESTEEM model. HPLC: high performance liquid chromatography; NIR: near-infrared; PADs/aPADs: paper analytic devices/antibiotic paper analytical devices.

The ESTEEM model simulated sampling, testing, and removal of substandard and falsified medicines from the Kenyan market using the four screening technologies and HPLC. The agent-drug in the model represents amoxicillin treatments used to manage pneumonia among children under age five in Kenya [18,19]. The flow of agent-drug through the simulation model is illustrated in **Fig 1**. Based on a previous study, 13 brands of amoxicillin were identified to be circulating on the Kenyan market [20]. Two brands were identified as having some substandard and falsified products (**Table 1**) [20]. Agents (amoxicillin treatments) were assigned a brand and given the characteristic of being either substandard/falsified or standard quality based on this market data.

**Table 1:** ESTEEM model inputs and parameters for Kenya.

Variables	Unit	Value	Standard Errors or Uncertainty ranges	Source
**Epidemiologic & Demographic**			** **	** **
Population under 5	Thousand	6,997	-	UN DESA [21]
Life expectancy	Year	61.10	-	UNICEF [22]
GDP per capita per day	USD	$3.99	-	World Bank [23]
Pneumonia incidence for children under 5	%	3.40	2.64–4.23	O’Brien et al. [24]
Pneumonia cases prescribed with amoxicillin	%	80	70–90	Authors’ assumption
Hospitalization	%	61.98	-	Nair et al. [25]
Number of patients				
National hospital	%	24	3.00	Ayieko et al. [26]
Provincial hospital	%	20	3.00	Ayieko et al. [26]
District hospital	%	23	3.00	Ayieko et al. [26]
Mission hospital	%	31	3.00	Ayieko et al. [26]
Average length of stay				
National hospital	Day	8.20	2.05	Ayieko et al. [26]
Provincial hospital	Day	6.60	1.65	Ayieko et al. [26]
District hospital	Day	5.42	1.35	Ayieko et al. [26]
Mission hospital	Day	6.09	1.52	Ayieko et al. [26]
Case-fatality ratio				
Hospital	%	3.90	0.80	Nair et al. [25]
Community	%	9.20	2.30	WHO [1]
**Costs**				
Diagnostic costs				
National hospital	USD	$8.89	31.92	Ayieko et al. [26]
Provincial hospital	USD	$1.68	6.36	Ayieko et al. [26]
District hospital	USD	$3.50	4.72	Ayieko et al. [26]
Mission hospital	USD	$11.69	10.39	Ayieko et al. [26]
Treatment costs per day				
National hospital	USD	$0.88	28.75	Ayieko et al. [26]
Provincial hospital	USD	$0.47	8.67	Ayieko et al. [26]
District hospital	USD	$0.26	2.64	Ayieko et al. [26]
Mission hospital	USD	$1.63	9.37	Ayieko et al. [26]
Cost per hospital day				
National hospital	USD	$8.30	10.78	Ayieko et al. [26]
Provincial hospital	USD	$6.46	4.86	Ayieko et al. [26]
District hospital	USD	$4.54	3.57	Ayieko et al. [26]
Mission hospital	USD	$4.56	4.20	Ayieko et al. [26]
Cost of Amoxicillin per tablet	USD	$0.01	0.00125	Calculated based on UNICEF’s report [18]
Extra treatment time with SF amoxicillin	Days	5	-	Authors’ assumption
Discount rate	%	3	-	Authors’ assumption
Discounted lifetime productivity losses	USD	$24,311	-	Calculated from GDP/capita, life expectancy, with 3% discount rate [22,23]
GDP per capita	USD	$1,455.4	-	World Bank [23]
**Amoxicillin Market Share**				
Number of brands	n	13	-	Myers et al. [20]
Number of batches per brand on the market	n	36	-	Author’s assumption
Batches exiting the market each month	n	1 per brand	-	Authors’ assumption
New batches entering the market each month	n	1 per brand	-	Authors’ assumption
Market share proportion and brand specific percentage of SF		**Market Share (% SF)**	** **	** **
Brand 1	n (%)	0.08 (0)	-	Myers et al. [20]
Brand 2	n (%)	0.24(0)	-	
Brand 3	n (%)	0.06 (11)	10%*	
Brand 4	n (%)	0.01 (0)	-	
Brand 5	n (%)	0.13 (0)	-	
Brand 6	n (%)	0.03 (0)	-	
Brand 7	n (%)	0.01 (0)	-	
Brand 8	n (%)	0.01 (0)	-	
Brand 9	n (%)	0.06 (0)	-	
Brand 10	n (%)	0.06 (0)	-	
Brand 11	n (%)	0.24 (59)	8%*	
Brand 12	n (%)	0.01 (0)	-	
Brand 13	n (%)	0.06 (0)	-	

GDP: gross domestic product; SF: substandard and falsified; UNICEF: United Nations Children’s Fund; UN DESA: United Nations Department of Economic and Social Affairs; USD: United States dollar; WHO: World Health Organization.

* Standard error was estimated for brand specific substandard and falsified proportions.

The model simulates sampling of medicines over a three-month time period during which 40 samples of 100 pills are selected at random from each of the 13 brands (520 samples in total) [20,27]. Screening of all the samples using a screening technology occurs based on the time per device stated in **Table 2**. After the screening, confirmatory testing is simulated to take one month during which time all samples that failed screening plus 10% of those samples not failing the screening undergo HPLC. Confirmed failed samples then undergo full pharmacopeial testing, taking 3 months. Finally, all batches linked to the failed samples are identified by the batch number and removed from the market. Implementing this process takes 9 months in total for PADs/aPADs, Raman, and NIR, 10 months for GPHF Minilab, and 19 months for HPLC on its own. This was run over a three-year period, where the sampling and testing with each technology occurred three times, while the scenario of HPLC on its own occurred only once.

**Table 2:** Device-specific parameters.

Variable	Devices					Source
	**HPLC**	**PADs/aPADs**	**GPHF-Minilab**	**NIR**	**Raman**	
**Screening**						
Sensitivity %	100	100/97	77.6	59.2	57.1	Amoxicillin Monograph [19], Weaver et al. [28], Myers et al. [20], ADB [17]
Specificity %	100	100/92	100	100	100	
Base cost of device USD (SE*)	$67,000 (6,700)		$4,361 (1,090)	$1,433 (358)	$64,141 (6,414)	Expert opinion, USP Technology Review [29], ADB [17]
Additional material cost per sample screened USD (SE*)	$47 (11.75)	$2/$2 (0.75)	$7.13 (1.78)	$0.04 (0.01)	$0.04 (0.01)	Expert Opinion, ADB [17]
Pills needed for each sample tested (n)	100	1/1	1	1	1	KPPB [27], Weaver and Lieberman [30], Authors’ assumption
Number of weeks for testing samples (weeks)**	12	2/2	7.5	1	1	ADB [17]
Number of devices (n)	1	1 per pill tested	1	1	1	ADB [17]

ADB: Asia Development Bank; HPLC: High performance liquid chromatography; KPPB: Kenya Pharmacy and Poisons Board; NIR: Near-infrared; PADs/aPADs: Paper analytic devices/antibiotic paper analytical devices.

*Standard error was calculated based on 25% of the mean.

**Estimated based on the testing time per sample [17].

To estimate the health and economic benefits of each screening scenario, we tracked the effects of using different screening devices for poor-quality antibiotics in terms of their impact on pediatric pneumonia outcomes. For each month of the model, the prevalence of substandard and falsified antibiotics was recorded and applied to the monthly cases of pediatric pneumonia. Pneumonia cases that received substandard/falsified versions of amoxicillin experienced double the case fatality rate and longer duration of illness. Detailed methods are described in the previous work [14].

The costs of implementing each screening technology as well as the costs of pediatric pneumonia cases are tracked throughout the model. The base cost of each screening device was added only once. Costs of treatment and productivity losses were calculated based on care-seeking, duration of treatment, and deaths due to pneumonia [21–23,26]. A discount rate of 3% was applied on future productivity losses.

Six scenarios were simulated. The base case scenario simulated no intervention, allowing the natural flow of poor-quality medicines through the market. Four scenarios simulated the use of screening technologies (PADs/aPADs, GPHF-Minilab, Raman, and NIR) prior to HPLC confirmation. The final scenario simulated the use of HPLC on its own to test every sample. These five scenarios were compared to the base case scenario to calculate outcomes. The primary outputs of the model were the costs of implementing each scenario and the benefits resulting from its implementation in terms of substandard and falsified treatments removed from the market, deaths averted, and costs including productivity losses averted. The costs per benefit were calculated as well as the return on investment for each screening scenario.

A probabilistic sensitivity analysis was conducted to capture uncertainties around our estimates. Gamma distributions were assumed for cost inputs, and beta distributions for probabilities and prevalence of substandard and falsified medicines. This paper presents averages over 10,000 model runs with the 90% uncertainty range (UR), a range that captures 90% of the simulated outputs.

### Results

**Fig 2:**
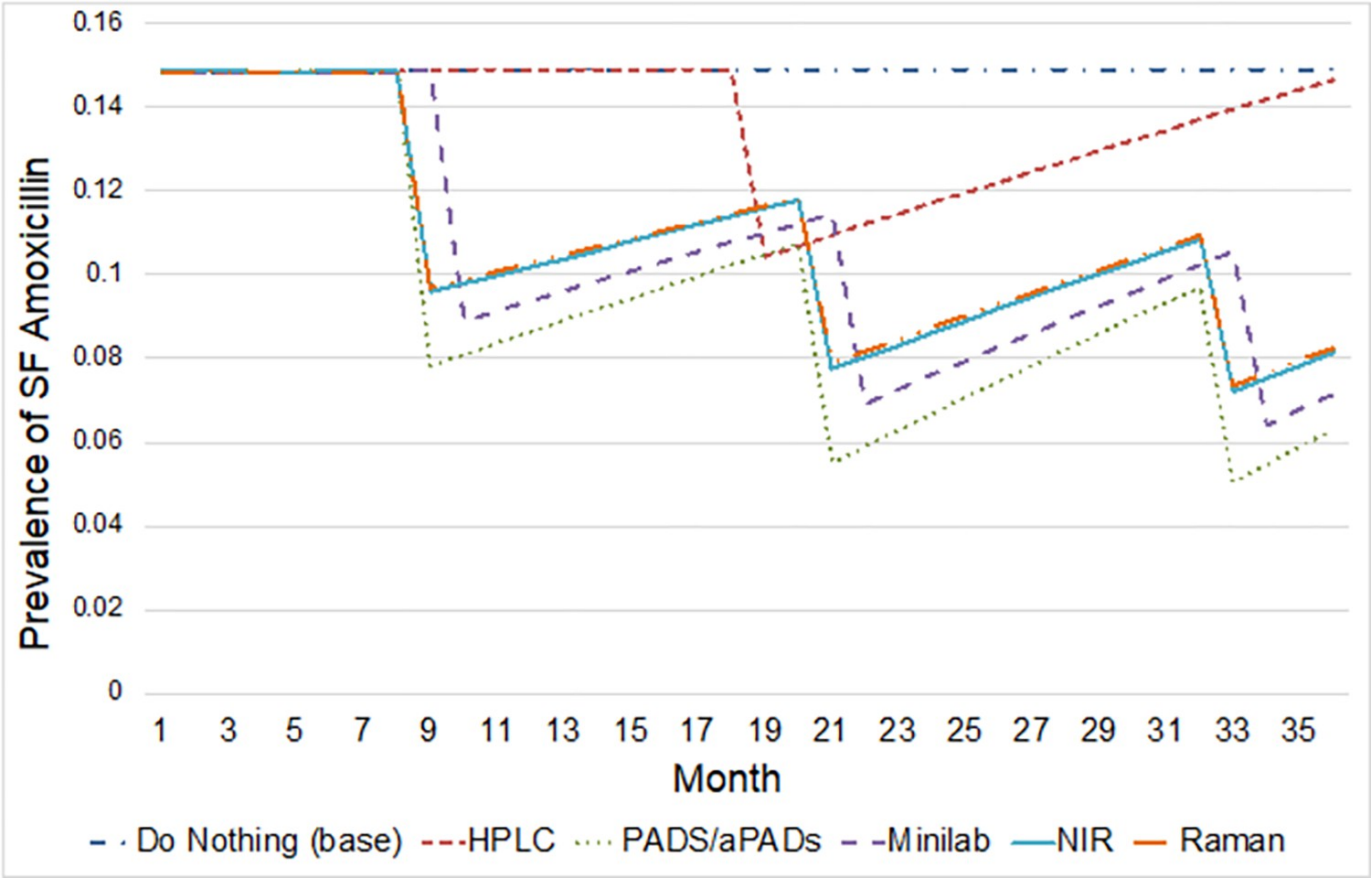
Monthly prevalence of substandard and falsified medicines over three years. HPLC: High performance liquid chromatography; NIR: Near-infrared; PADs/aPADs: Paper analytic devices/antibiotic paper analytical devices.

The monthly prevalence of substandard and falsified medicines over three years for each scenario is compared in **Fig 2**. In the base case scenario, no poor-quality amoxicillin was removed from the market, resulting in a stable amount of substandard and falsified amoxicillin available to patients, as indicated by the straight line in **Fig 2**. Testing amoxicillin products using HPLC took the longest time, which meant that substandard and falsified amoxicillin was left to circulate until the removal period at month 19. Consequently, by the time of removal many of the batches identified as substandard/falsified were already utilized by patients or had expired and left the market. For the HPLC scenario, an annual average of 3,188 (90%UR 1,642–4,819) substandard and falsified amoxicillin treatments were removed, while all other scenarios removed more than double that amount (**Table 3**).

**Table 3:** Average annual costs, benefits, and return on investment of screening technologies.

	Do Nothing (base)	HPLC	PADs/aPADs	GPHF-Minilab	NIR	Raman
**Costs**						
Costs of Screening with Intervention	$0	$35,069	$20,974	$23,115	$16,544	$37,444
**Benefits**						
Treatment costs	$13,907,757	$13,809,652	$13,459,781	$13,546,077	$13,564,787	$13,571,686
Productivity losses						
Short-term	$5,457,100	$5,412,766	$5,254,860	$5,293,716	$5,302,168	$5,305,354
Long Term	$313,987,426	$310,796,340	$299,438,958	$302,232,344	$302,846,063	$303,070,305
Incremental benefits excluding productivity		$98,105	$447,976	$361,681	$342,971	$336,072
Incremental benefits including productivity		$3,333,526	$15,198,684	$12,280,147	$11,639,266	$11,404,939
**Cost per Benefit**						
Number of child pneumonia deaths	12,915	12,784	12,317	12,432	12,457	12,466
Deaths averted		131	598	484	458	449
Cost per death averted		$267	$35	$48	$36	$83
Average substandard and falsified treatments received by patients	28,378	26,156	18,245	20,192	20,616	20,775
Average substandard and falsified treatments averted from use		2,222	10,133	8,186	7,762	7,603
Cost per substandard and falsified treatment averted		$15.79	$2.07	$2.82	$2.13	$4.92
Average substandard and falsified treatments removed from market	0	3,188	11,613	10,213	9,124	8,945
Cost per treatment removed		$11.00	$1.81	$2.26	$1.81	$4.19
**Return on investment**						
ROI excluding productivity losses		$3	$21	$16	$21	$9
ROI including productivity losses		$95	$725	$531	$704	$305

HPLC: High performance liquid chromatography; NIR: Near-infrared; PADs/aPADs: Paper analytic devices/antibiotic paper analytical devices; ROI: Return on investment.

Of the four screening technologies, PADs with aPADs were the most effective at identifying poor-quality amoxicillin, as demonstrated by the steeper drop in months where removal takes place (**Fig 2**). The PADs/aPADs scenario saw the highest annual removal of poor-quality amoxicillin treatments (**Table 3**, n = 11,613, 90%UR 7,777–15,627). Similarly, GPHF-Minilab, NIR, and Raman were all effective at identifying substandard and falsified amoxicillin. However, due to the longer duration of sample testing with GPHF-Minilab, the removal of medicines in that scenario occurred a month later than the other three screening devices (**Fig 2**). Increases in prevalence seen between removal months were the result of the natural influx of substandard and falsified medicines in the market as new batches were added each month and old ones expired (**Fig 2**).

The total cost of implementing post-market surveillance with a screening technology included costs for sampling, testing, and then removal of amoxicillin. Annual sampling costs were estimated at $11,965 (90%UR $7,489 - $17,107) for three months of sampling in each of the three years for PADs/aPADs, NIR, Minilab, and Raman. For HPLC, only one three-month sampling period was included, resulting in a three-year cost of sampling of $11,965. Costs of personnel to remove substandard/falsified amoxicillin were estimated at $997 (90%UR $624 - $1,425) annually across the four portable screening technologies. Total annual cost to implement these technologies was lowest for NIR at $16,544 (90%UR $11,502 - $22,311), followed by PADs/aPADs ($20,974, 90%UR $15,388 - $27,315), GPHF-Minilab ($23,115, 90%UR $17,171 - $29,865), and Raman ($37,444, 90%UR $31,307 - $44,188). The estimated total costs to test all samples with HPLC was $35,069 (90%UR 30,075–40,509) annually. The base cost of the Truscan Raman at $64,141, was comparable to that of the HPLC reported at $67,000 (**Table 2**). The other devices had lower up-front costs with PADs/aPADs purchased at $2 per test strip each, NIR at $1,433, and the GPHF-Minilab at $4,361. The annual cost of screening for substandard and falsified amoxicillin using NIR was always lower than other devices at an average of $5,429 (90%UR $4,310 - $6668). Annual average screening costs were comparable for PADs/aPADs and GPHF-Minilab at $20,974 (90%UR $15,383 –$27,315) and $23,115 (90%UR $17,171 –$29,865), respectively. The annual average cost of using the portable Raman device ($37,444, 90%UR $31,307 - $44,188) was more expensive than that of HPLC ($35,069, 90%UR $30,075 - $40,509) owing to their high initial cost and because the Raman was used to screen three times over the three-year period compared to once for HPLC (**Table 3**).

The estimated average number of substandard/ falsified treatments and the number of deaths averted with the implementation of various screening technologies and HPLC is presented in **Table 3**. Without any screening measures in place, on average 28,364 (90%UR 20,045–36,707) substandard and falsified amoxicillin treatments were simulated to be administered to children under age five, resulting in an estimated 12,970 (90%UR 9,375–17,024) deaths annually in Kenya. With the implementation of screening technologies, fewer substandard and falsified treatments reached patients. The simulation showed that by screening with PADs/aPADs 10,137 (90%UR 6,604–13,786) fewer pneumonia cases were treated with poor-quality amoxicillin. GPHF-Minilab reduced the administration of poor-quality amoxicillin by 8,184 treatments (90%UR 5101–11,386), NIR by 7,761 treatments (90%UR 4,675–11,033), and Raman by 7,575 treatments (90%UR 4,554–10,742). The cost of averting one substandard/falsified treatment from being used by a patient was lowest using NIR at $0.70, which was comparable to PADs/aPADs ($1.00) and GPHF-Minilab ($1.25). The cost per substandard and falsified treatment averted by Raman and HPLC were higher at $3.48 and $14.11, respectively. Compared to the base case scenario, the average number of deaths averted per screening technology was highest for PADs/aPADs with 598 (90%UR 351–897) deaths averted, followed by GPHF-Minilab (484, 90%UR 272–735), NIR (458 (90%UR 250–708) and Raman (449, 90%UR 249–696). Average costs per death averted were low for PADs/aPADs ($35, 90%UR $21 - $62), NIR ($36, 90%UR $20 - $69), and GPHF-Minilab ($48, 90%UR $28 - $88) scenarios, compared to $83 (90%UR $51 - $152) for Raman and $267 (90%UR $155 - $618) for HPLC.

**Fig 3:**
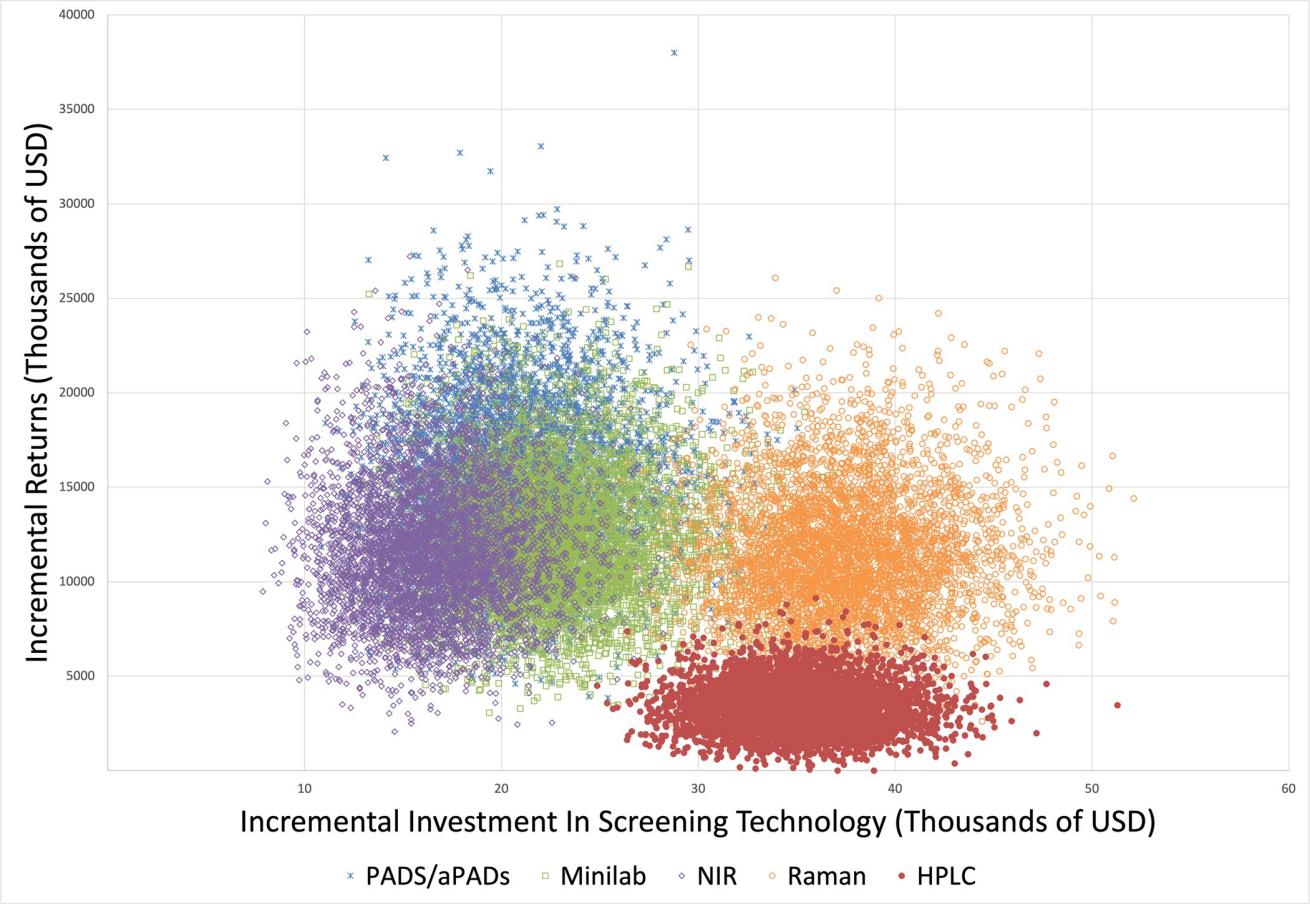
Incremental costs and benefits per screening technology. HPLC: High performance liquid chromatography; NIR: Near-infrared; PADs/aPADs: Paper analytic devices/antibiotic paper analytical devices. *Incremental returns include both long- and short-term productivity losses. Each scenario is calculated in comparison to the base case of doing no post-market surveillance.

In the base case scenario, in which substandard and falsified amoxicillin was left to circulate on the market freely, annual direct treatment costs were estimated at approximately $13 million (90%UR $4.5 - $30 million) for childhood pneumonia (**Table 3**). Short- and long-term productivity losses of pediatric pneumonia were estimated at $5 million (90%UR $4.3 –$6.8 million) and $315 million (90%UR $228 - $414 million), respectively. Implementing PADs/aPADs, GPHF-Minilab, NIR, or Raman was estimated to save between $11.4 million to $15.2 million in direct treatment costs and averted productivity losses annually. All screening devices and HPLC were found to have a high return on investment. The highest returns on investment were associated with utilization of NIR at $21 (90%UR $5 –$51) and PADs/aPADs also at $21 (90%UR $6 –$51) per dollar invested, owing to the low cost of both devices. For every dollar invested, we estimated an expected return of $3 (90%UR $0 - $6) for HPLC, $9 for Raman (90%UR $2 –$21), and $16 (90%UR $4 –$38) for GPHF-Minilab (**Table 3**). HPLC and Raman were estimated to have similar incremental costs, but Raman exhibited increasing benefits over time due to its ability to identify and subsequently remove substandard and falsified amoxicillin much faster (**Fig 3**).

## Discussion

The health and economic impact of different screening technologies was estimated in the context of post-market surveillance of amoxicillin in Kenya. The study results indicate that PADs/aPADs, GPHF-Minilab, NIR, and Raman are very similar in their abilities to quickly identify substandard and falsified amoxicillin and can have a substantial impact when used to narrow down which poor-quality medicines to remove from the market. We show that speeding up testing using any screening device results in greater returns than using HPLC on its own. Fewer substandard and falsified amoxicillin treatments were simulated to be administered to children under age five for pneumonia when implementing screening before HPLC, resulting in fewer annual deaths. Overall, all screening technologies resulted in a positive return on investment indicating that interventions to screen for substandard and falsified products are a worthwhile economic investment.

Despite their similarities, these screening technologies vary in terms of device acquisition costs, personnel training needs, testing costs, and time requirements. Fast, reliable, and low-cost drug screening tools are essential for increasing the detection of substandard and falsified medicines in LMICs [5,31]. The NIR is a field-portable device that uses dispersive spectroscopy to identify the API without destroying the sample or requiring any sample preparation [12,31]. In this study, the NIR yielded a higher ROI than using only HPLC owing to the lower device cost and much lower testing costs. A previous study compared the implementation of six screening devices in Laos, finding that with a high prevalence of substandard and falsified antimalarials, implementing any device was cost-effective, with NIR found to be most cost-effective [32]. The present analysis adds to the existing literature, finding NIR to have one of the highest ROIs in a different LMIC setting and for a different medicine class.

Raman and GPHF-Minilab screening technologies also yielded higher ROI than using only HPLC to screen amoxicillin samples. The Truscan Raman spectrometer is a nondestructive field-portable device that employs a dispersive spectroscopy technique analogous to the NIR system [11,17]. The acquisition of the Truscan Raman may seem cost-prohibitive for some regulatory authorities in LMICs, as they are about as expensive as the initial investment in HPLC. However, it can be utilized as an alternative to HPLC for expedited screening of medicine quality, and according to our simulations would be quicker than using HPLC to screen all samples. The GPHF-Minilab, deemed as a ‘lab-in-a-suitcase’, is a field-portable device employing disintegration and semi-quantitative thin layer chromatography (TLC) technique that has been extensively and successfully used to detect substandard and falsified medicines in LMICs [12,17,33,34]. This study demonstrated that the GPHF-Minilab has a relatively high ROI, further supporting its continued use for post-market surveillance in LMICs.

PADs and aPADs are newer, inexpensive paper-based colorimetric test cards that have been developed to screen for poor-quality antibiotics [20,28–30]. In our simulations, PADs/aPADs identified the most substandard and falsified antibiotics. This is due to the high sensitivity and specificity of utilizing PADs along with the associated antibiotic-specific aPADs which can be used to detect substandard amoxicillin [20]. A recent study demonstrated that utilization of PADs/aPADs for screening poor-quality amoxicillin in Kenya resulted in faster removal of substandard and falsified products and higher annual incremental benefits compared to using HPLC alone [14].

Costing results should be utilized in conjunction with a broader understanding of the environment where these screening devices will be implemented. For example, one study found that users wrongly categorized antimalarial samples more often when using PADs compared to using other screening devices [35]. We chose to evaluate screening devices that would be specifically suited to use in LMICs in that they do not require electricity or a special laboratory setup. Some other important factors to consider are the training level of the personnel conducting testing, the speed of the tests, and whether quantitative (level of API) or qualitative results (existence of specific API) are needed for the evaluation [10]. The underlying prevalence of falsified compared to substandard medicines can also be an important consideration, as all screening devices have been found to be less cost-effective in scenarios of low prevalence [32]. Using a combination of these screening devices could also be an effective strategy. A recent study conducted in Ghana demonstrated the usefulness of combining three low cost (<USD $10,000) screening methods (GPHF-Minilab, colorimetry, and Counterfeit Drug Indicator) to quickly detect substandard and falsified artemether/lumefantrine products [36].

Utilizing HPLC on its own yielded the lowest ROI compared to the four screening technologies, despite being able to accurately identify all substandard or falsified medicines. This is owing to the amount of time that HPLC requires to test individual samples. During that time, poor-quality batches remain on the market where they are likely to be used by patients. Despite the differences between these screening technologies, the model estimates that doing some post-market surveillance, even with the most expensive options, still yields a return on investment by averting deaths and costly hospital care.

HPLC is regarded as the gold standard for analyzing API. Most pharmacopeias, including the United States, European, and British Pharmacopeias, utilize it for compendial analysis of pharmaceutical products to establish acceptable quality standards [13,37]. However, given the high costs and resources needed to conduct HPLC, a stepwise three-level approach has been proposed for medicine quality control in LMICs. This involves (1) visual/physical inspection first, followed by (2) simple, rapid, and cost-effective screening tests such as TLC and spectroscopic technologies, and finally (3) confirmatory pharmacopeial analysis (e.g. HPLC) [38]. This analysis provides evidence for the return on investment of strengthening this second level of quality control involving screening technologies. Kenya has two WHO prequalified Quality Control Laboratories with HPLC capability for confirmatory pharmacopeial tests: the Ministry of Health’s National Quality Control Laboratory (NQCL) and Laboratory of the Mission for Essential Drugs and Supplies (MEDS) [39]. Incorporating screening technologies can considerably reduce the burden of compendial testing in these laboratories and the cost burden on the regulatory authority.

Sustainable funding of national medicine regulatory agencies is crucial for strengthening and expanding current post-market surveillance capability in Africa [40]. While national regulatory agencies largely bear the costs of implementing post-market surveillance activities, the net benefits accrued have far-reaching health and economic impacts [40]. This study should serve as a call to action for national medicine regulatory authorities to shift current post-market surveillance practices from initial HPLC analysis to the stepwise three-level approach. Particularly, implementation of initial field screening using simple, reliable, and fast technologies can be used to expedite post-market surveillance and lower its total cost [15]. For successful adoption of these technologies, national governments should prioritize passing country-specific legislation to facilitate swift and appropriate response to medicines failing screening tests [41]. Further research is still needed to fill the gaps in the literature on the capabilities, costs, and benefits of screening technologies to inform post-market surveillance decisions in more LMICs.

This study has some key limitations. First, there was limited reliable and quality data for Kenya. The ESTEEM model fails to capture the geographical heterogeneity of Kenya in that assumptions were made for a variety of inputs based on local or regional data. For instance, for brand market share, data from a study in Western Kenya was used [20]. An estimate for the case fatality rate of childhood pneumonia deaths for children under age five across Africa was employed [24,25]. It was also assumed that personnel testing amoxicillin products across the country have the same level of training, and therefore, the specificity and sensitivity of devices would be relatively uniform. The study team utilized the most recent reliable data sources available at this time by conducting a thorough literature search. Second, in estimating the ROI of using a select screening and testing device, it was assumed that once a substandard and falsified medication is confirmed with HPLC, the associated batch was removed from the market before patient consumption. Third, this paper primarily focused on a single use of amoxicillin for the treatment of pneumonia among children without considering its other indications. Therefore, the cost and benefit outcomes are largely conservative. Furthermore, while this was a case study in Kenya, the results might vary based on country-specific regulatory and quality control capabilities, ultimately affecting the selection of screening technology. The type of medicine tested using a specific screening technology also affects the sensitivity, specificity, cost, and overall impact to be realized. Further analysis with more inclusive data across countries is warranted.

## Conclusions

This study highlights screening technologies with a higher ROI than the current HPLC standard for medicine quality control. While HPLC largely remains the standard instrument for confirmatory testing, other technologies can be used to reduce costs and speed up the removal of poor-quality medicines, and consequently improve health and economic outcomes. Regulatory authorities should use this data to strengthen their surveillance efforts. This will enable respective government ministries of health to save costs both directly and indirectly through improved health from the consumption of quality medicines.
